# Slowing of axonal regeneration is correlated with increased axonal viscosity during aging

**DOI:** 10.1186/1471-2202-11-140

**Published:** 2010-10-25

**Authors:** Phillip L Lamoureux, Matthew R O'Toole, Steven R Heidemann, Kyle E Miller

**Affiliations:** 1 Department of Zoology, Michigan State University, East Lansing, MI 48824-1115, USA; 2 Department of Mathematics, Michigan State University, East Lansing, MI, 48824-1115, USA; 3 Department of Physiology, Michigan State University, East Lansing, MI 48824-3320, USA

## Abstract

**Background:**

As we age, the speed of axonal regeneration declines. At the biophysical level, why this occurs is not well understood.

**Results:**

To investigate we first measured the rate of axonal elongation of sensory neurons cultured from neonatal and adult rats. We found that neonatal axons grew 40% faster than adult axons (11.5 µm/hour vs. 8.2 µm/hour). To determine how the mechanical properties of axons change during maturation, we used force calibrated towing needles to measure the viscosity (stiffness) and strength of substrate adhesion of neonatal and adult sensory axons. We found no significant difference in the strength of adhesions, but did find that adult axons were 3 times intrinsically stiffer than neonatal axons.

**Conclusions:**

Taken together, our results suggest decreasing axonal stiffness may be part of an effective strategy to accelerate the regeneration of axons in the adult peripheral nervous system.

## Background

Following injury of peripheral nerves in adults, significant regeneration occurs but at a rate slower than in the young [1]. For example, using radiotracer studies Pestronk et al., [2] found the average rate of regeneration of rat sciatic sensory neurons occurs at a rate of ~2.6 mm/day in animals that are 2 mo old and slows to a rate of 0.3 mm/day in animals that are 28 mo old. Based on these numbers regeneration of a nerve with a length of 1 m could be accelerated from ~8 years to ~1 year if rates of regeneration found in younger animals could be achieved in adults. If we understand the mechanics of axonal elongation, it could be possible to devise strategies to speed regeneration of peripheral nerves from years to months, allowing the reinnervation of distal muscles before the occurrence of irreversible loss of muscle function [3,4].

While the molecular influences (inhibitory proteins, growth factors, adhesion molecules, etc.) underlying poor regeneration of adult peripheral neurons have been extensively analyzed [5,7], the intrinsic biophysical properties of individual neurons have only recently been investigated [8-14]. Our recent work using embryonic sensory neurons suggests that axonal elongation occurs through a two step process where forces at the growth cone stretch the axon and new material is added along the axonal shaft [15,16]. Our mathematical modeling predicts the rate of axonal stretching/elongation is a function of the level of force generation at the growth cone, the strength of adhesions of the axon to the substrate, axonal diameter, and the mechanical stiffness (i.e. viscosity) of the axon [11]. To determine if there are intrinsic biophysical differences that could explain the slow regeneration of adult sensory neurons, we used force calibrated towing needles to characterize the biophysical properties of neonate rat and adult rat axons. We found no difference in the adhesion levels between the neonate and adult rat neurons, but a significant difference in axonal viscosity that increased with developmental age.

## Results

### The rate of axonal elongation decreases with aging

**Figure 1:**
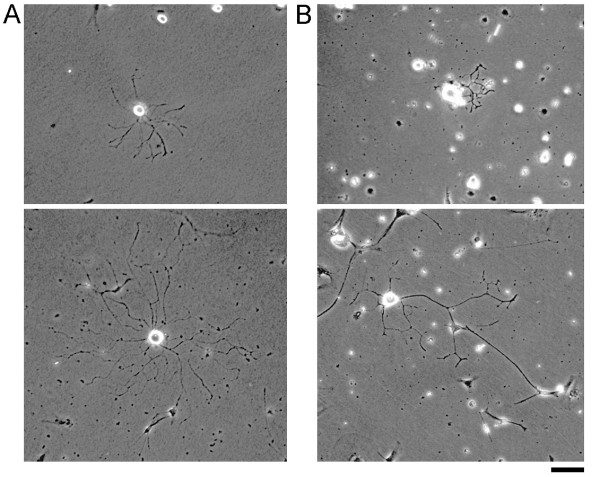
**Examples of the outgrowth of neonatal (A.), and adult sensory neurons (B.)**. The top panels show growth after 1 day and the bottom panels show growth after 2 days in culture; bar = 45 µm.

We chose to focus our analysis of aging on the biophysics of outgrowth of sensory neurons from neonatal and adult rats because their outgrowth is well characterized at the molecular and cellular levels [17,18]. This system has been of interest because adult sensory neuron axonal regeneration is enhanced by prior 'conditioning' lesions, which activate and inactivate the expression of groups of proteins linked to embryonic regeneration [7,19-21]. Figure 1 shows an example of the outgrowth of unconditioned neonatal and adult sensory neurons. We found, as has been previously reported [17], that the elongation of adult sensory neurons was characterized by extensive axonal branching and a relatively slow rate of outgrowth (8.2 +/- 1.1 µm/hour; average +/- 95% confidence interval (CI)). Neurons from neonatal animals, also exhibited extensive branching, but had a significantly higher rate of axonal elongation than neurons from adult animals (11.5 +/- 1.4 µm/hour; average +/- 95% CI). These data are fully summarized in Table 1. The rates of growth were significantly different with p < 0.05 using a two tailed *t*-test. These results confirm the premise that the rate of axonal regeneration decreases during aging.

**Table 1:** The rate of axonal regeneration decreases with age

Age	Average (µm/hour)	s.d.	# of growth cones	n	s.e.m.	95% CI
embryonic	35	19	12	42	2.93	5.9

neonate	11.5	11.7	34	268	0.72	1.4

adult	8.2	6	32	118	0.55	1.1

Note the embryonic data were previously published [16]

### The axons of adult sensory neurons lengthen by stretching

**Figure 2:**
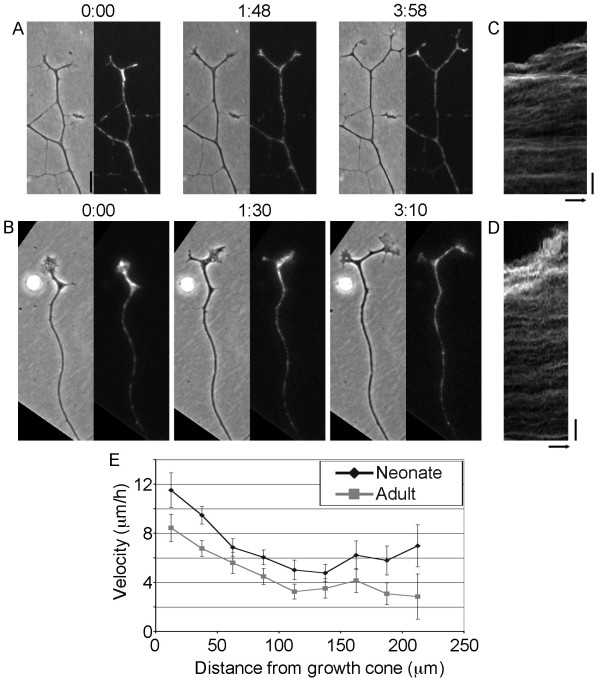
**Adult and neonatal sensory neurons elongate by stretching, but at a slower rate than embryonic neurons**. Matched phase images of axons and fluorescent images of mitochondria during normal growth of (A.) neonatal rat, and (B.) adult rat sensory neurons. C & D. The kymographs (bar 20 µm, arrow 1 hour) illustrate the advance and spreading of mitochondria along the axons in each type of neuron (see Additional files 1 and 2). E. A quantitative comparison of low velocity mitochondrial transport. The error bars in the graphs are the 95% confidence intervals.

To determine how the mechanism of axonal elongation changes during aging, we stained the mitochondria along the axons and monitored the movement of the docked mitochondria during normal growth cone mediated axonal elongation (Figure 2). In the sensory neurons from neonatal and adult animals, we found that axonal elongation was coupled with axonal stretching, but with quantitative differences in the velocity profiles (Figure 2E, see Additional files 1 and 2). Analysis of the velocity profiles, reveals that the velocity of bulk movement along the neonatal axons was consistently higher as compared to the adult axons. The differences observed here suggested that sensory neurons from neonatal and adult neurons regenerate in vitro by the same fundamental mechanism (i.e. stretch and intercalated mass addition along the axon) as we previously reported for embryonic neurons [11,15,16]. It raised the question of whether the slower rate of growth of the adult neurons was due to a difference in axonal diameter, adhesion strength to the substrate, axonal viscosity, or force generation at the growth cone.

### Axonal viscosity increases during aging

**Figure 3:**
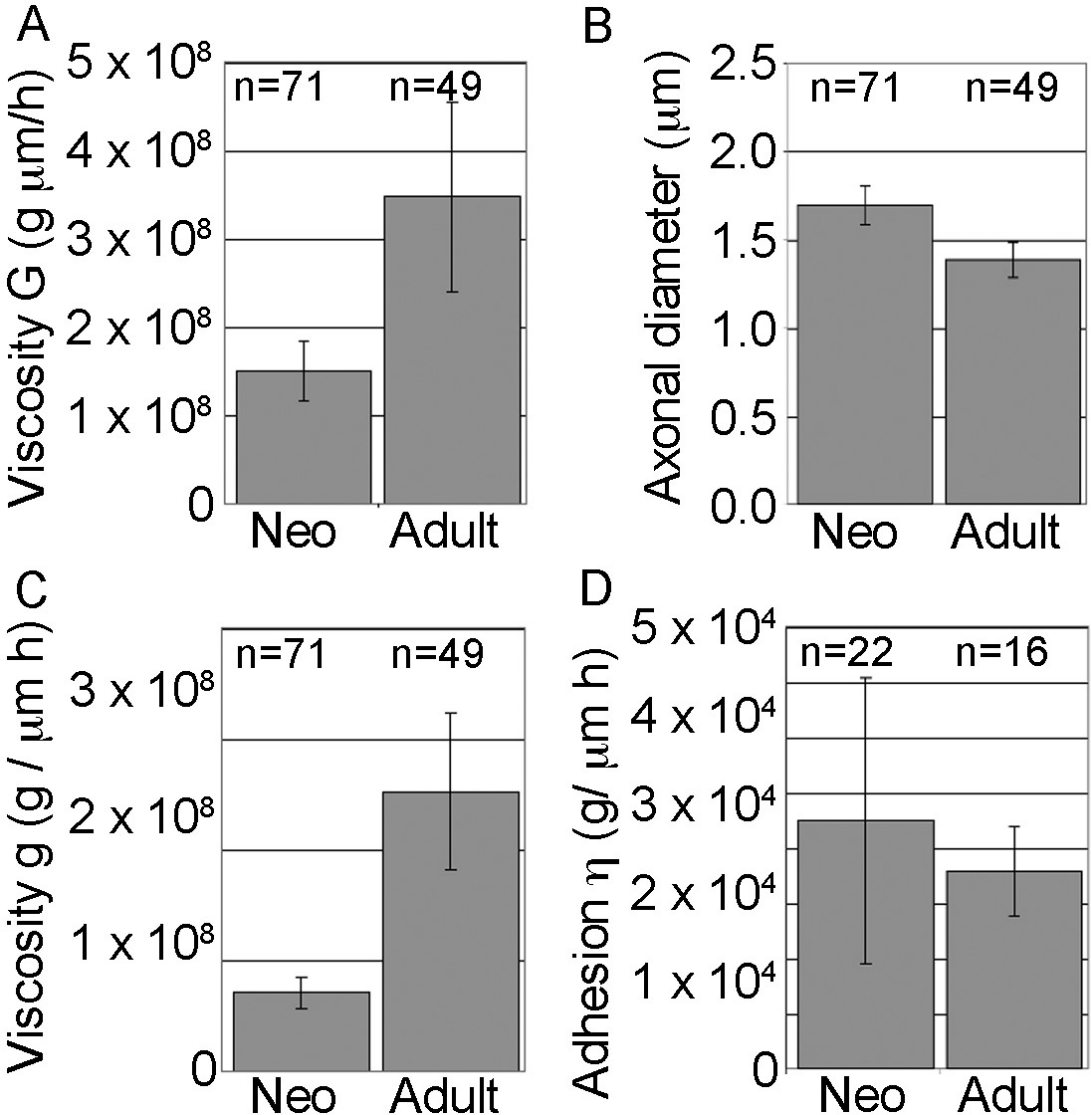
**Axonal viscosity increases during aging**. A. Axonal viscosity (*G*) which is an aggregate measure of axonal diameter and intrinsic viscosity (g) increased as a function of age and was significantly different between all groups. B. Axonal diameter decreased as a function of age. C. The intrinsic viscosity (g), increased significantly with aging. D. The strength of adhesions (η) was similar in neurons from neonatal and adults. For all graphs, the average +/- 95% CI is shown and the number of measurements is listed above the bars.

To determine the viscosity and the strength of adhesions, we applied known levels of forces to growth cones to experimentally stimulate elongation and analyzed the movement of docked mitochondria to measure the biophysical properties of neurons as previously described [11]. We found that the overall viscosity (*G*) of the neurons from adult animals was significantly higher (p < 0.0001, two-tailed *t*-test) by a factor of 2 than neurons from neonatal animals (Figure 3A). In contrast, there was a small but significant decrease (p < 0.001, two-tailed *t*-test) in axonal diameter (Figure 3B). Because *G *is a function of axonal diameter, the underlying differences in the intrinsic viscosities (*g*) were even larger. The *g *value of the adult neurons was 4× higher (p < 0.0001, two-tailed *t*-test) than the neonatal and adult neurons (Figure 3C). We did not observe significant differences (p = 0.4, two-tailed *t*-test) in the levels of adhesion between the neonatal and adult neurons (Figure 3D). Together these results suggested that a substantially higher intrinsic viscosity was the key biophysical difference between the neonatal and adult neurons.

### Growth cone force generation is not reduced during aging

One possibility to explain the slower rate of adult axonal elongation is that the growth cones generate lower levels of force. To investigate, we estimated force generation at the growth cones of neonatal and adult neurons with Eq. 1.

(1) $$F_{0}=v\sqrt{G\eta }.$$

Taking the average velocities (*v*) of axonal elongation (Table 1) and using the values of *G *and η from Table 2, we found the level of force generation (F_0_) in the neonatal and adult growth cones to be 230 and 223 µdynes respectively (Table 3). As these data show the level of force generation in neonatal and adult growth cones is similar, a reduction in force generation seems unlikely to be the underlying factor that causes the reduction in regeneration during aging.

**Table 2:** Axonal biophysical parameters as a function of age

Age	Variable	Average	s.d.	n	s.e.m.	95% CI
embryonic	G (g µm/hour)	3.9E+07	3.0E+07	31	5.4E+06	1.1E+07
neonatal	G (g µm/hour)	1.5E+08	1.4E+08	71	1.7E+07	3.3E+07
adult	G (g µm/hour)	3.5E+08	3.8E+08	49	5.4E+07	1.1E+08
						
embryonic	diam (µm)	2.1	0.7	187	0.05	0.1
neonatal	diam (µm)	1.7	0.5	71	0.05	0.11
adult	diam (µm)	1.4	0.4	49	0.05	0.1
						
embryonic	g (g/µm hour)	1.3E+07	8.5E+06	31	1.5E+06	3.1E+06
neonatal	g (g/µm hour)	7.1E+07	5.9E+07	71	7.0E+06	1.4E+07
adult	g (g/µm hour)	2.5E+08	2.5E+08	49	3.5E+07	7.1E+07
						
embryonic	η(g/m hour)	9.6E+03	7.5E+03	28	1.4E+03	2.9E+03
neonatal	η(g/m hour)	4.5E+04	4.1E+04	22	8.7E+03	1.8E+04
adult	η(g/m hour)	3.6E+04	1.5E+04	16	3.8E+03	8.2E+03

Note the embryonic data were previously published [11]

**Table 3:** Estimated force generation at the growth cone as a function of age

Age	Velocity (g µm/h)	G (g µm/h)	(g/µm h)	F_0 _(g µm/h^2^)	F_0 _(nN)	F_0 _(µdyne)
neonatal	11.5	1.5E+08	4.5E+04	3.0E+07	2.3	230
adult	8.2	3.5E+08	3.6E+04	2.9E+07	2.2	223

Changes in sensory neuron growth cone size have been observed during embryonic development. In particular, growth cones from embryonic day 7 neurons grown on polylysine are 146% greater in size than neurons from embryonic day 14 neurons [22]. To determine if growth cone size is changing between postnatal and adult neurons, we measured their area. We found the average area of the neonatal growth cones to be 46 +/- 51 µm^2 ^(average +/- standard deviation, n = 53 growth cones) and the area of the adult growth cones to be 63 +/- 54 µm^2^(n = 46 growth cones). While there was a trend for adult growth cones to be larger, we did not find a significant difference in growth cone size using a two-tailed *t*-test.

## Discussion

As we age our bodies and minds become less flexible. Presumably, this is the result of changes that are occurring at the cellular level. To determine if there are intrinsic biophysical changes in individual neurons that could explain the slowing of axonal regeneration that occurs during aging, we used force calibrated towing needles to characterize the biophysical properties of neonate rat and adult rat axons. We chose this type of neuron because they have been extensively characterized in terms of growth and the molecular changes that occur during regeneration [1,7,17-21]. We found no significant differences in terms of axonal diameter, adhesion strength, or force generation by the growth cone, that could explain the slowing of axonal regeneration during aging. Yet we did find a significant increase in viscosity of the axons that increased with developmental age.

The conventional model of axonal elongation suggests that axonal elongation occurs primarily through a mechanism that involves the assembly of new axon at the tip of an otherwise stationary axonal framework [23,24]. In contrast, our recent work on the rapidly growing (i.e. ~35 µm/hour) embryonic chick sensory neurons suggests a Stretch and Intercalation model which proposes axonal elongation occurs through a two step process where forces generated at the growth cone stretch the axon and new material is added along the axonal shaft [11,15,16]. At a biophysical level, there are two simple possibilities that could explain the slowing of axonal regeneration that occurs during aging. The first is that the rate of elongation differs because the mechanism of growth differs (e.g. tip growth vs. stretch and intercalation) [11]. The second is that the mechanism of elongation is the same, but that rates of elongation differ because of quantitative differences in key parameters. To distinguish between these possibilities we stained the mitochondria along the axons and monitored the movement of the docked mitochondria during normal growth cone mediated axonal elongation (Figure 2). As we observed in the sensory neurons from embryonic animals [16], here we found that in neonatal and adult animals axonal elongation was coupled with axonal stretching, but with quantitative differences in the velocity profiles (Figure 2E). We take this as evidence that these neurons share the same basic mechanism of outgrowth but differ quantitatively in terms of their biophysical properties.

Based on our mathematical modeling, the slowing of elongation with aging could arise from an increase in the strength of adhesions (η) between the axons and the substrate, an increase in the diameter of the axons (d), an increase the intrinsic viscosity (*g*) of the axons, a decrease in the forces generated by the growth cones (*F_0_*), or some combination of these variables. To experimentally test our predictions based on growth cone mediated axonal elongation, we applied known levels of forces to growth cones and analyzed the movement of docked mitochondria to measure the biophysical changes in the neurons that were occurring during aging. These experiments strongly suggested that the key factor was not an increase in the strength of adhesions (η), but rather an increase in total axonal viscosity (*G*). To determine if the increase in *G *was the result of a change in axonal diameter or intrinsic axonal viscosity, we measured axonal diameter. We found that neurons cultured from older animals had a significantly smaller axonal diameter, which indicates that the intrinsic viscosity (*g*) of the axons is increasing. Based on our measurements of average rate of axonal elongation, axonal viscosity (*G*), and adhesion strength (η), we estimated the force generation at the growth cone (F_0_) for sensory neurons cultured from the neonatal and adult rats. This formula suggested that force generation was similar in these two type of neurons (Table 3). Altogether, the most conservative interpretation of our data is that the primary factor limiting the rate of elongation in the adult neurons is their high intrinsic viscosity (*g*).

A caution to be noted in interpreting the adhesion data (η) is that our experiments were conducted on neurons grown on polyornithine. *In vivo, *laminin and chondroitin sulfate proeteoglycans are modulated through development and upregulated after peripheral nerve injury [25-28]. In addition the receptors for laminin, integrins, are developmentally regulated and when expressed increase regeneration following axotomy [29-32]. While we did not observe changes in adhesion strength for neurons grown on polyornithine as a function of age (Figure 3), such changes may be occurring. Further analysis involving systematic analysis of adhesion on physiologically relevant substrates is needed and may reveal significant differences in substrate adhesion during neuronal maturation.

As we have previously conducted a biophysical analysis of the elongation of chicken embryonic sensory neurons [11], it is of interest to compare the results published here with our previous work. Nonetheless it is important to keep in mind that differences between the properties of the chicken sensory neurons with the rat sensory neurons could be attributed to species or age. With these considerations, in our measurements of axonal viscosity we found both total axonal viscosity (*G*) and intrinsic axonal viscosity (*g*) increased substantially with the developmental age of the animal (Table 2). Statistical analysis between the published and new data, revealed significant difference between all three groups (p < 0.0001 for each, two-tailed *t*-tests). This suggests there is a progressive increase in axonal stiffness through the embryonic, neonatal, and adult developmental stages.

A previous analysis of the cellular changes that are occurring in growth cones during developmental maturation demonstrated that a significant (> 50%) decrease in sensory neuron growth cone size occurs between embryonic day 7 and 14 [22]. This is correlated with a decrease in the sensitivity of axonal elongation to cytochalasin and an increase in the stability of the microtubule and neurofilament cytoskeletons. While we did not observe significant differences in growth cone size between neonatal and adult sensory neurons (46 +/- 51 µm^2 ^vs. 63 +/- 54 µm^2 ^respectively), our results are on balance consistent with the findings of Jones et al., [22]. In particular, our observation that axonal viscosity increases with age could be explained by increased stability of neurofilaments, as they observed following treatment with cytochalasin; and/or reduced microtubule dynamics, as they observed based on levels of tyrosinated tubulin and EB3 comet velocity [22].

**Figure 4:**
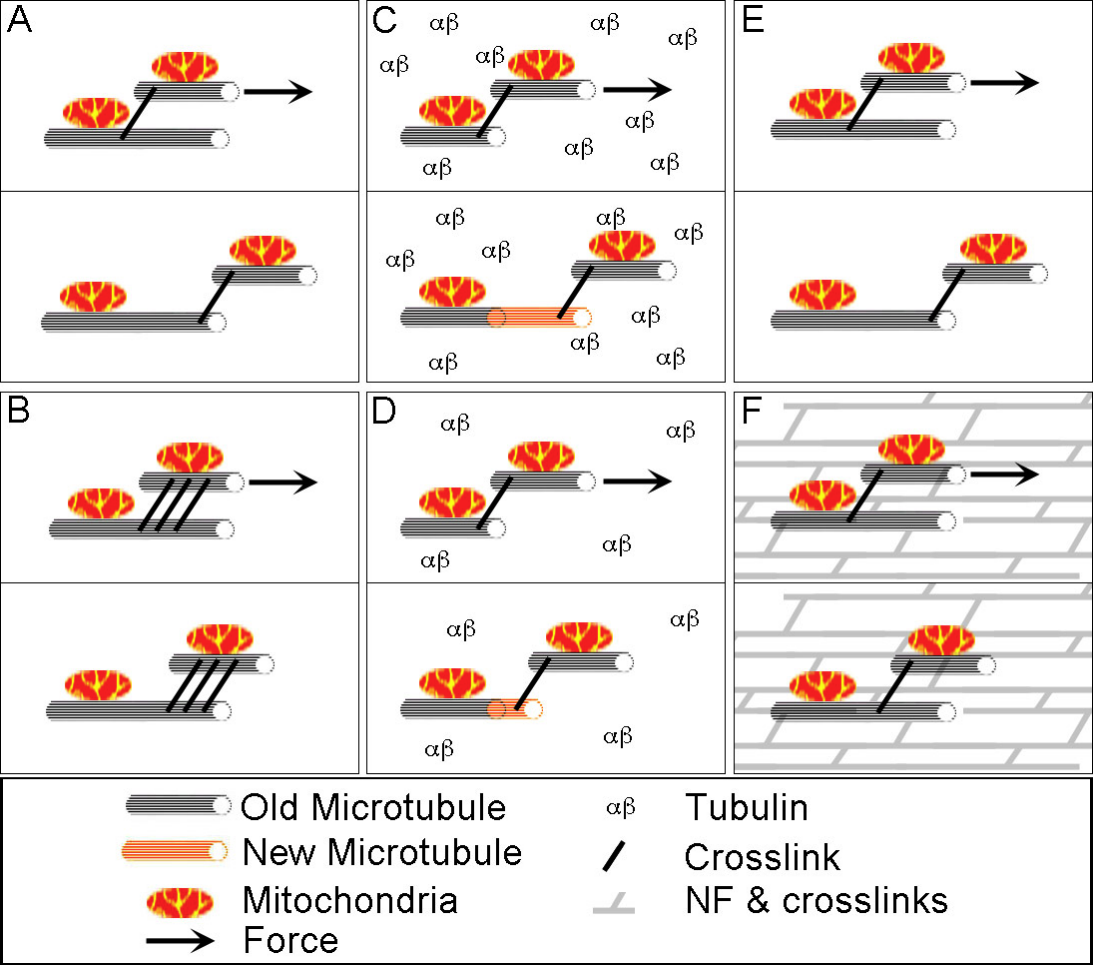
**Hypothetical roles of viscosity in axonal elongation**. A. Microtubules with docked mitochondria are shown interacting through cross linkers. B. The presence of additional or different types of cross-linkers could increase axonal viscosity in adult axons. C. Microtubule polymerization coupled with microtubule sliding. D. A reduction in the concentration of tubulin could limit axonal growth. E. The relative absence of neurofilament polymers in embryonic axons could reduce axonal viscosity as compared to F. adult axons.

Our reported differences in axonal viscosity are likely to reflect changes in the expression patterns of genes involved in axonal elongation and regeneration [6,7,18,23]. Viscosity is minimally a complex function of molecular events such as sliding of cross-linked polymers, microtubule and actin assembly in response to tension, and membrane addition to the plasma membrane [10,33]. During periods of rapid elongation associated with development and regeneration, tubulin expression is increased and neurofilament expression is decreased [20,34]. How changes in tubulin expression might change axonal viscosity are at present unknown, but as it has been shown that the application of forces to non-neuronal cells induces microtubule polymerization [35], increasing the availably of tubulin may act to decrease the amount of force needed to lengthen the axon (Figure 4C and 4D). In contrast, reduction of neurofilament expression may decrease axonal viscosity [36-40] (Figure 4E and 4F). Finally, there are changes in the expression of MAPs and tau that are correlated with development and regeneration following axotomy in sensory neurons [21,41-44]. Changes in the composition of cytoskeletal associated proteins could contribute to altered assembly dynamics and cross-linking (Figure 4A and 4B), either of which could increase axonal viscosity in adult neurons. Axonal regeneration involves changes in a multitude of molecular pathways [5,7,34,45-48], a major challenge for the future is to link specific proteins to cellular biophysical parameters.

## Conclusions

Our work is the first to examine the biophysical changes that occur in individual neurons during aging. We found adult axons grew ~30% slower and were 3 times intrinsically stiffer than neonatal axons. Taken together our results suggest targeted molecular approaches to decreasing axonal stiffness may be part of an effective strategy to accelerate the regeneration of axons in the adult peripheral nervous system.

## Methods

### Cell culture

Adult and neonate sensory neurons were cultured by a modification of a protocol developed by Lindsay et al., [17,49], which uses a gentle enzymatic dissociation of the ganglion tissue. Supernumerary adult rats (> 200 g) were euthanized and their DRGs dissected and placed into Hanks Balanced Saline, without Ca^2+ ^and Mg^2+^, buffered to pH 7.4 with 5 mM HEPES (HBSS-). After removal of ventral roots and ganglia capsules, the DRGs were dissociated for 10 minutes at 37°C in activated papain (Worthington Biochemical Corp, Lakewood, NJ) at a concentration of 50 U/ml in HBSS-. The ganglia from adult animals were transferred to a second enzyme solution containing 5 mg/ml dispase: 1 mg/ml collagenase (Life Technologies, Carlsbad, CA) in HBSS- for 10 minutes at 37°C, these enzymes were not used for the neonates. The ganglia were then triturated 10 times with a fire-polished Pasteur pipette, followed by another 10 min digestion and trituration in the same solution. Single cells were separated from larger chunks by gravity sedimentation. The single cells were then pelleted by brief centrifugation, and resuspended in culture medium for plating. Axonal outgrowth was supported in L-15 containing 10% fetal calf serum and 50 ng/ml 7S nerve growth factor (NGF) and N9 growth supplement [50] in plates treated with 0.01% polyornithine.

### Mitochondrial labeling

To track axonal stretching during normal axonal elongation and while towing, mitochondria were labeled with Mitotracker [11] and observed with a Leica DM IRB inverted microscope and observed with a N Plan L 40/0.55 corr Ph2 with an adjustable collar infinity/0 - 2/c objective. Cells were illuminated with a 100 W Xenon lamp attenuated 98% with neutral density filters through a Texas Red cube (Chroma, Rockingham, VT) for visualization of MitoTracker [11].

### Data analysis

Images were taken with Openlab (Improvision, Waltham, MA) using an Orca-ER digital camera CCD, model #CA742-95 (Hamamatsu, Bridgewater, NJ), converted into TIFFs and analyzed using ImageJ (NIH) as previously described [11].

### A biophysical model of axonal elongation

**Figure 5:**
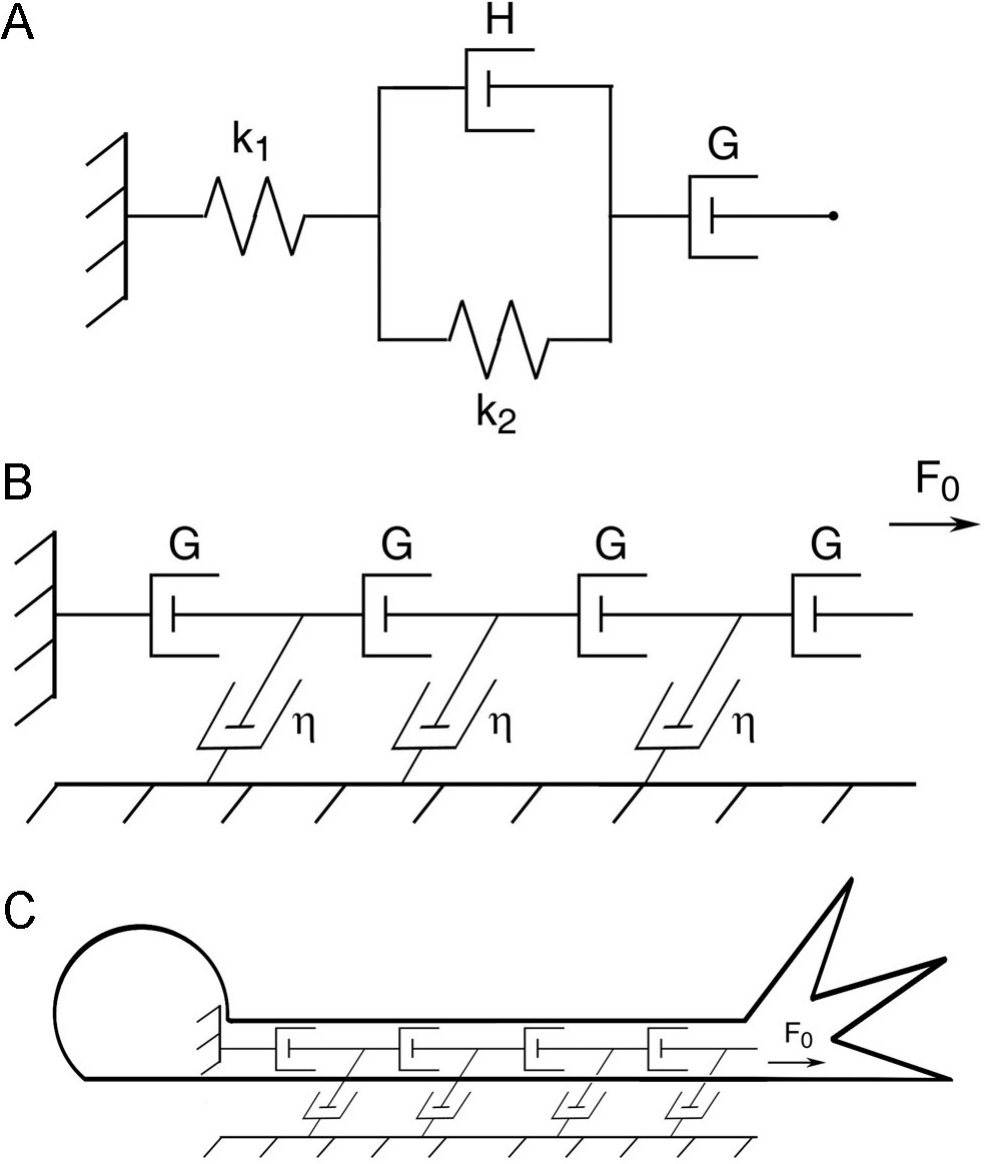
**Biophysical model of an axon**. A. A single Burgers element with a free spring (k_1_); a spring (k_2_) and dashpot (H) in parallel; and a free Growth dashpot (G). B. Under constant tension (F_0_) the behavior of each Burgers element is dominated by its free dashpot, thus we treat the axon as a series of dashpots (G). Attachments to the substrate are represented as friction dashpots (η). C. Tension applied or generated in the growth cone (F_0_) is dissipated by interactions with the substrate along the axon.

Based on our prior work, axonal elongation in response to forces acting at the growth cone occurs in three stages [51]: after an initial elastic stretch, there is delayed stretching, followed by elongation at a constant rate. This behavior is described by a three-element model consisting of a spring (k_1_), a spring (k_2_) and a dashpot (H) in parallel, and a dashpot (G) in series (Figure 5A). A dashpot is a mathematical construct for viscosity and obeys the relationship *force = viscosity constant velocity. *In our model [11], we treat the axon as a series of viscoelastic elements that interact with each other and the substrate (Figure 5B). Since the elastic components of the model are invariant under steady state conditions (such as elongation over the course of hours) we simplify the Dennerll model to a series of dashpots. Two factors that determine the velocity profile of an axon under tension are the axon's axial viscosity (*G*) and the constant of friction (η) that quantifies the interactions between the axon and the substrate (Figure 5C). Both of these parameters characterize resistance to flow and have dimensions of viscosity. The axial viscosity *G *is the amount of force needed to distend a unit amount of axon at unit velocity and is a function of intrinsic axonal viscosity (*g*) and the cross sectional area of the axon (*A*). Increasing axonal diameter has the effect of adding dashpots in parallel or, equivalently, increasing the dashpot constant. If an axon alters its diameter as a result of stretching or mass addition, but maintains its physiological properties, then varies while is unchanged. The coefficient of friction is characterized by the strength and the number of adhesions between axon and substrate (Figure 5C). Such adhesions have been shown to have effects on both axonal stretching and growth cone advance [52]. η is assumed to be zero where the axon is unattached to the substrate and increases when adhesions form or strengthen. Our mathematical model, based on the elongation of embryonic sensory neurons, has been previously described in detail.

(2) $$v\left[x,L(t)\right]=\frac{F_{0}\sinh\left[x{\left(\eta /G\right)}^{1/2}\right]}{{\left(\eta G\right)}^{1/2}\cosh\left[L(t){\left(\eta /G\right)}^{1/2}\right]}.$$

In brief, Eq. 2 gives the profile for velocity as a function of distance, *x*; axonal length, *L*; force at the growth cone, *F_o_*; axonal viscosity, *G*; and adhesion strength, η. For the details on the derivation of this formula refer to [11].

### Determination of axonal viscosity and adhesiveness

Axons were towed as previously described [11]. In brief, force calibrated towing needles previously coated in polylysine (1 mg/ml) and concanavalin A (1 mg/ml) were used to apply forces to growth cones. Force measurements were acquired from phase images throughout the experiment. To track bulk movement along the axon in response to forces, fluorescent images of docked mitochondria were analyzed using ImageJ (NIH). To calculate *G *and η, lines were fitted to the velocity data in the kymographs to calculate the rate of change of the velocity of the mitochondria. Using force measurements from the calibrated needles, a value of was found by dividing the average force over this interval by the slope of the fitted line. Once values of were determined (one value of per 30 minute) the Origin software package (OriginLab Corporation, Northampton, MA) was used with Eq. 2 to fit the best value of η to the data. For this calculation the velocities of mitochondria proximal to the point of adhesion were used. Empirical values of *F_o_*,*L *, and *G *were fixed and a Levenberg-Marquardt algorithm was implemented in the Origin package to find the optimal value of η. The relationship *G = gA (A =*cross-sectional area) was used to calculate the intrinsic axial viscosity *g *for each axon. Phase images of each trial were analyzed using ImageJ to determine the axonal diameter at various times. For each phase image, the diameter was measured along the axon as described below.

### Axonal width measurement

To automate the process of axonal diameter measurement, we developed an ImageJ plugin (named 'Width Measurement') that measures pixel intensity across objects, finds the derivative to determine the steepest points, and then calculates the distance between these points [11]. The plugin has previously been described in detail [16]. In brief, the plugin uses the derivative of pixel intensity across the axon to find these steepest points and returns the distance between these points to calculate axonal width. To prepare images for analysis, images of axons acquired at 12-bit pixel depth were opened in ImageJ, converted to 32-bits, and straightened using the "Straighten"; plugin [53]. The straightened images were then stretched 8× on the y-axis by interpolation using the ImageJ plugin TransformJ set to quintic B-spline [54]. To remove high frequency noise, a Gaussian Blur filter with a radius 2 pixels was applied using the built-in ImageJ function. The 'Width Measurement' plugin was then run to determine axonal width at each pixel along the axon. The source code for the Plugin is available on request.

### Growth cone area measurement

To determine the area of growth cones, the width and length of individual growth cones were measured from phase images using the line tool in ImageJ. These numbers were then multiplied to give growth cone area. While our goal was to simply determine if there is a change in growth cone size, we note that because growth cones are not perfectly square, the calculated numbers for growth cone area are an overestimate.

All animal studies were approved by the Michigan State University Institutional Animal Care and Use Committee.

## Competing interests

The authors declare that they have no competing interests.

## Authors' contributions

PL acquired the data for all of the experiments, MO analyzed the data to determine the biophysical coefficients, SH - helped conceive the study, developed the protocol for isolating adult DRG neurons, and edited drafts of the manuscript, KM - helped conceive the study, designed the experiments, and wrote the manuscript. All authors read and approved the final manuscript.

## Supplementary Material

Additional file 1 **Movement of the axonal framework occurs during normal axonal elongation of neonatal sensory neurons**. This movie demonstrates that mitochondria docked to the axonal framework advance forward during normal axonal elongation. Sensory neurons from neonatal rats were grown on plastic dishes, coated with poly-L-ornithine, for 2-3 days and then were stained with MitoTracker Red CMX-Ros (0.1 µM) on the day of imaging. Fluorescent images were acquired every 2 minutes to visualize mitochondrial distribution and phase images were acquired at 15 to 30 minute intervals to resolve axonal morphology. The time of image acquisition for the phase and fluorescent images is shown as h:min in the movie. The movies were straightened to follow the right axonal branch using the Straighten plugin in ImageJ. A kymograph illustrating the forward advance of mitochondria, stably docked to the axonal framework, is shown on the right hand side of the movie. The kymograph was generated from the straightened image. The length of the arrow above the kymograph represents 1 hour and the vertical scale bar is equal to 20 µm. The dot that moves across the top of the kymograph denotes the time position in the movie. The movie is displayed at 20 frames per second. Click here for file

Additional file 2 **Movement of the axonal framework occurs during normal axonal elongation of adult sensory neurons**. Sensory neurons from adult rats were grown, labeled, and observed using the same conditions as the neurons from neonatal animals. The time of image acquisition for the phase and fluorescent images is shown as h:min in the movie. The movies were straightened to follow the right axonal branch using the Straighten plugin in ImageJ. A kymograph illustrating the forward advance of mitochondria, stably docked to the axonal framework, is shown on the right hand side of the movie. A kymograph illustrating the forward advance of mitochondria, stably docked to the axonal framework, is shown on the right hand side of the movie. The kymograph was generated from the straightened image. The length of the arrow above the kymograph represents 1 hour and the vertical scale bar is equal to 20 µm. The dot that moves across the top of the kymograph denotes the time position in the movie. The movie is displayed at 13 frames per second. Click here for file
